# Performance of Circulating Placental Growth Factor as A Screening Marker for Diagnosis of Ovarian Endometriosis: A Pilot Study 

**DOI:** 10.22074/ijfs.2015.4606

**Published:** 2015-12-23

**Authors:** Cinzia Zucchini, Paola De Sanctis, Chiara Facchini, Nadine Di Donato, Giulia Montanari, Valentina Bertoldo, Antonio Farina, Alessandra Curti, Renato Seracchioli

**Affiliations:** 1 Department of Experimental, Diagnostic and Specialty Medicine, University of Bologna, Bologna, Italy; 2 Pelvic Endoscopy and Minimally Invasive Gynaecologic Surgery, St. Orsola Malpighi University Hospital, Bologna, Italy; 3 Department of Medicine and Surgery DIMEC, Division of Prenatal Medicine, St. Orsola Malpighi Hospital, University of Bologna, Bologna, Italy

**Keywords:** Endometriosis, PlGF, Blood Marker, Endometrioma

## Abstract

**Background:**

The aim of this study is to compare the circulating placental growth factor (PlGF) concentration in women with and without endometrioma to verify the performance of this marker to diagnose the disease.

**Materials and Methods:**

In this case-control study, thirteen women with histological diagnosis of ovarian endometriosis were compared with women without endometriosis disease. PlGF plasma levels of endometriotic patients and controls were investigated using a fluorescence immunoassay technique.

**Results:**

PlGF showed a direct correlation with body mass index (BMI) only in the control group (P=0.013). After adjustment for BMI values, PlGF median value in endometriosis group (14.7 pg/mL) resulted higher than in control group (13.8 pg/ mL, P=0.004).

**Conclusion:**

PlGF is a promising peripheral blood marker that can discriminate between patients with and without ovarian endometriosis.

## Introduction

Endometriosis is a estrogen-dependent chronic disorder often resulting in morbidity, pelvic pain and infertility (1). Although endometriosis typically appears benign on histological examination, it is characterized by a malignant tumour-like nature in that it grows, infiltrates and adheres to the surrounding tissues. The gold standard for diagnosis is laparoscopic surgery with histologic confirmation. However, every surgical procedure has potential risks for patients (2). Ultrasound should be the first-line imaging modality for the evaluation of patients with suspected endometriosis. Its accuracy has greatly improved over recent years, but its performance is heavily operator-dependent (3). Detection of simple and non-invasive diagnostic test is one of the priorities in endometriosis research. 

Most of the proposed non-invasive diagnosis methods are based on the identification of biomarkers believed to be involved in the pathophysiology of the disease and differentially expressed in the peripheral blood of patients as compared to health subjects. The increasing interest in angiogenetic factors as putative peripheral blood markers for endometriosis is not surprising, since several lines of evidence suggest that the angiogenetic factors are involved in the establishment of neovascularization requirement for development and maintenance of endometriosic lesion (4,8). 

Vascular endothelial growth factor (VEGF) is the most widely studied angiogenetic factor. Although several authors have evaluated serum or peripheral blood levels of VEGF in endometriosis patients, contradictory results have been reported and the validity of using VEGF in endometriosis diagnosis has not been definitely attested (9,17). 

Placental growth factor (PlGF) is a member of the proangiogenic vascular endothelial growth factor family (18,19). PLGF presented some similarities to the structure of VEGF-A with a 42% amino acid sequence identity. Nevertheless, they have significant functional differences. PlGF was originally identified in the placenta, where it has been proposed to control trophoblast growth, differentiation and invasion (20,22). Its biological effect is mediated by VEGF receptor 1 (VEGFR-1), a tyrosine kinase receptor expressed on the surface of several cell types including endothelial cells, macrophages, bone marrow precursors and cancer cells (23). PlGF shows a high disease-specific activity and its contribution to the angiogenetic switch in pregnancy, wound healing, ischemic conditions and tumor growth has been well documented (24,30). In cancer, PlGF may also facilitate metastasis by increasing the motility and invasion of malignant cells, and it has been demonstrated that the levels of PlGF in plasma and serum correlate with tumor stage and poor survival in various tumors (31,37). Despite the above-mentioned angiogenetic and prometastatic activities, the role of PlGF as candidate biomarker in diagnosis of endometriosis has been poorly investigated. Suzumori et al. (38) have indicated increased levels of placental growth factor in the peritoneal fluid of women with endometriosis compared with women with cystadenomas, suggesting that the production of PlGF may contribute to the pathogenesis of endometriosis by promoting neovascularization. 

We aim to analyze and to compare the preoperative PlGF concentration in the peripheral blood of women with and without ovarian endometriosis in order to verify the performance of this putative marker to diagnose the disease. 

## Materials and Methods

### Subjects

**Fig.1:**
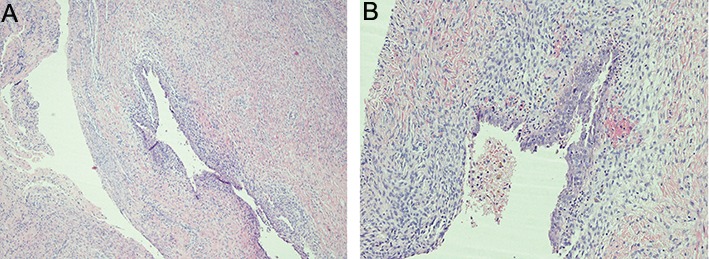
Histology of an ovarian endometriosis cyst wall showing endometrial tissue in the muscular layer with granulation tissue. A. Haematoxylin and eosin (H&E) staining method (×4 magnification) and B. The cyst wall is lined by cylindrical endometrial-type epithelium. (H&E) staining method (×10 magnification).

In the present case-control study, we enrolled a total of 26 women in our center of Pelvic Endoscopy and Minimally Invasive Gynaecologic Surgery, S. Orsola-Malpighi University of Bologna, Bologna, Italy, from October 2012 through September 2013.Ethics Committee approval of S. Orsola-Malpighi Hospital was obtained before starting the data collection. The approval code is PLGF 167/2012/O/Tess.All study subjects provided a written informed consent for the use of biological specimens for research purposes. Thirteen patients (group A) with a preoperative ultrasound diagnosis of ovarian endometriosis (defined as the presence of a unilocular cyst with a regular wall and homogeneous low-level echogenicity of the cyst’s content), subsequently confirmed by histological analysis (Fig .1), were included in the study. For each case, a consecutive control of same age, parity and BMI without endometriosis disease was recruited; therefore, a 1:1 match was generated. Patients of the control group (group B) were operated for leiomyoma pathology. Again, we excluded primi/ pluriparae women with history of pre-eclampsia and/ or intrauterine fetal growth restriction (IUGR). All patients were submitted on peripheral blood collection during the proliferative phase of the menstrual cycle and they were not on hormonal treatment since at least 3 months (Table 1). 

### Blood samples/measurement of placental growth factor concentration

**Fig.2:**
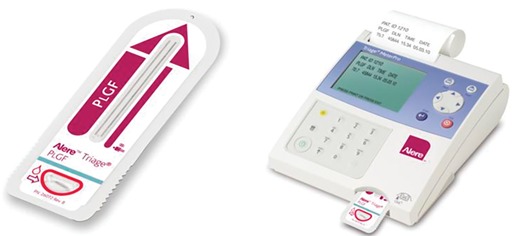
Placental growth factor (PlGF) measurement instrument.

A peripheral blood sample (10 ml) from each woman enrolled in our study was collected in sterile tubes containing ethylenediaminetetraacetic acid (EDTA) and treated for PlGF evaluation within 2 hours of being drawn. Blood samples were centrifuged at 1500 g for 10 minutes at 4˚C; the obtained plasma samples were stored at –20˚C until the measurement of PlGF plasma levels. PlGF quantification was performed by the Alere PlGF Test (Fig .2) using Triage^®^ MeterPro instrument (Alere Srl, Italy), according to the manufacturer’s instructions. The test is based on a fluorescence immunoassay technique and provides a PlGF measurable range of 12 to 3,000 pg/mL. 

**Table 1:** Demographic and clinical characteristics of the data set


Variable	Controls(n=13)	EndometriosisCases(n=13)	Pvalue*

Median age (minimum-maximum)(Y)	34.5(25-46)	34(26-45)	0.513
Previous surgery			
None	76.9	46.2	0.303
Laparoscopic surgery	0	7.7	1.00
Abdominal surgery	23.1	30.8	1.00
Both	0	15.4	0.485
Nulliparity(%)	91.7	53.8	0.073
Cysts			
Right side% (medianmm)	-	7.7(28.5mm)	-
Left side%(medianmm)	-	53.8(36.0mm)	-
Bilaterality(%)	-	38.5	-
Median BMI (minimum-maximum)	22.1(18.8-29.7)	22.3(19.6-31.1)	0.572
Median PlGF (minimum-maximum)(pg/mL)	13.8(13.7-18.6)	14.7(14.5-21.0)	0.004


*; Mann-Whitney U test or Fisher exact test, BMI; Body mass index and PlGF; Placental growth factor.

### Statistical analysis

Descriptive statistics was performed by means of routine analysis. Adjustment for possible confounding variables was performed by means of a general linear model (GLM) having PlGF as dependent variable plotted versus any possible correlated variable. Mann-Whitney U test and Chi square or Fisher exact test were used to compare the two generated groups of patients. Finally a non -parametric Receiver Operator Characteristics (ROC) curve was generated in order to calculate the sensitivity of adjusted PlGF values for endometriosis at fixed rate of false positive. A two tails P<0.05 was considered statistically significant. 

## Results

**Fig.3:**
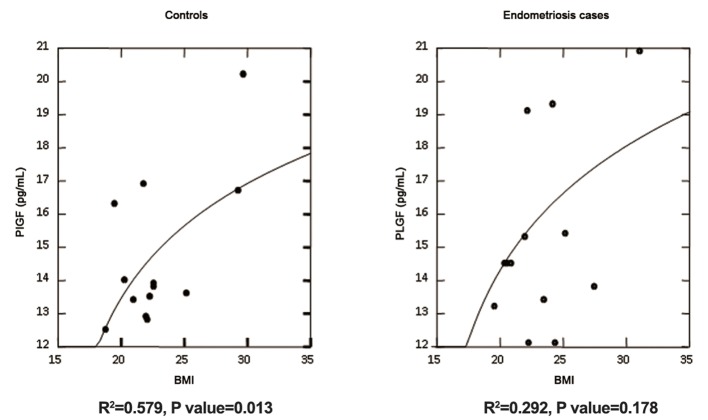
Log-Linear regression of BMI vs. PlGF in controls and cases. As shown significant association has been found only for controls (P=0.013). For endometriosis cases, PlGF lost its significant correlation with BMI (P=0.178). BMI; Body mass index and PlGF; Placental growth factor.

PlGF showed a direct correlation with body mass index (BMI), but only in the control group (P=0.013). For endometriosis group, in fact, PlGF lost its significant correlation with BMI (P=0.178) as showed in figure 3. After adjustment for BMI values and using the parameters of regression model quoted for the controls, the PlGF in endometriosis group resulted in a slight higher median value when compared to that of the control group (P=0.004) as reported in table 1. A non-significant direct correlation was found between PlGF and parity and it was excluded from the final model. There was no difference for all the other variables considered in the data set as reported in table 1. 

**Fig.4:**
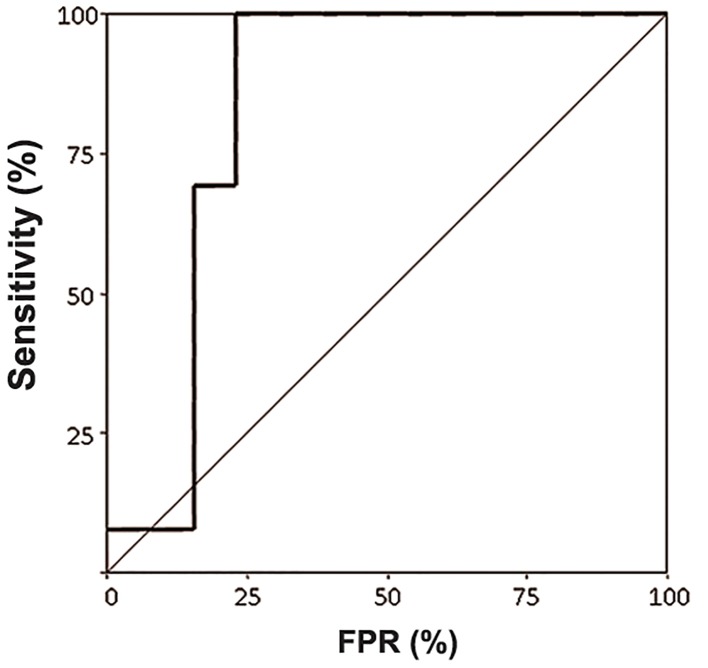
Receiver operator characteristics (ROC) curve for detection of endometriosis using placental growth factor (PlGF) as explorative variable. The sensitivity of PlGF for endometriosis was 80% at a fixed false positive rate (FPR) of 20% about, with an area under the curve (AUC)=0.834 (95%CI=0.649-1.020) and P=0.004.

ROC curve yielded a sensitivity of PlGF for endometriosis of 80% at a fixed false positive rate (FPR) of 20% about, with an area under the curve (AUC)=0.834 (95%CI=0.649-1.020) and a P=0.004 (Fig .4). 

## Discussion

To date, the gold standard for diagnosis of endometriosis is laparoscopic surgery (2). This limitation, together with the long delay between the onset of symptoms and diagnosis of endometriosis, is the main reasons for the urgent require of a noninvasive diagnosis method. 

Ultrasound is also accurate, but only if performed by an expert operator (3). Detection of simple and non-invasive diagnostic test is one of the priorities in endometriosis research. 

The identification of peripheral blood markers, capable of diagnosing or excluding endometriosis, could avoid the need for an invasive procedure (39,40) or at least allow symptomatic women to be screened. Nevertheless, a biomarker with high sensitivity, specificity and clinical relevance useful for non-invasive diagnosis of endometriosis, is still unidentified. 

From the pathophysiological point of view, it has been demonstrated that the ectopic implants of endometrial cells are rich in angiogenetic growth factors and it is well known that the establishment of a new blood supply is crucial for the development of endometriotic lesions. Taking into account the importance of angiogenesis in the pathogenesis of the disease (7), in this study, we assessed the role of PlGF, a member of the proangiogenic VEGF family, as putative circulating biomarkers of endometriosis. 

We demonstrated that PlGF correlates with BMI in controls as a possible biological epiphenomenon of some tissue release. Whereas in endometriosis patients, this direct association is lost probably for a secondary confounding effect due to the presence of the disease. After adjustment for BMI, in fact, the PlGF resulted higher in ovarian endometriosis patients. Again, even if the PlGF increase in endometrioma is quite small, it is statistically significant by means of non-parametric analysis. Several authors have previously reported a direct association between BMI and both VEGF and PlGF plasma levels (41,44). In endometriosis patients, both actual PlGF values and correlation with BMI described in control subjects seems altered. Regardless of the mechanistic link between BMI and increased circulating angiogenetic factors, which has not been clarified yet, the impairment of the relationship between BMI and circulating PlGF suggests the involvement of this proangiogenic factor in endometriosis. It may be considered an indicator of the disease. 

Despite the small differences in PlGF values, a ROC curve yielded a significant AUC with a sensitivity of 80% about at FPR of 20%. The associated PlGF cut-off was 15 pg/mL. For the linear regression, given a sample size of 13 subjects, a power of 83% at a 5% of type I error is reached for a R2=0.6. Given the R2 found in this study (0.579 and 0.292 for controls and cases, respectively), the sample size required to reach a proper power was 17 and 87. Our samples reached instead a power of 64 and 17 at a 5% of type I error. For the ROC curve, instead, a sample size of 26 cases (13+13) and an AUC of 0.834 yielded a power of 95% at a type I error of 5%. Even if this is a small series of data, the results seem encouraging for a possible use of PlGF in ovarian endometriosis evaluation in prospective studies. An increasing number of reports has documented that PlGF activity does not affect quiescent vessels in healthy tissues, but it has a role in vessel stabilization under pathological conditions (24,27). This disease-restricted activity of PlGF is an attractive property that could help to discriminate between pathological and health conditions. However, the involvement of PlGF in many other angiogenetic diseases raises the question whether PlGF could be specific enough to be proposed as a marker of endometriosis and further explorations are needed to clarify this issue. 

A noteworthy feature of our study is that it employs a highly reproducible and easily-applied technique of PlGF quantification. This method is readily amenable, employs no toxic reagents and is very fast. These features make the procedure feasible in terms of clinical management and/or largescale screening. 

## Conclusion

Our study identifies PlGF level as a promising biological indicator that could help to discriminate between patients with ovarian endometriosis and healthy subjects. Further investigations are needed to explore PlGF specificity degree and to confirm its prognostic/diagnostic value in clinical practice. Nevertheless, our results support the possibility of finding an easily detectable peripheral blood marker that alone or within a panel of others biomarkers could improve the diagnosis of endometriosis in symptomatic women. 
